# Retrospective evaluation of treatment outcomes of Gasserian ganglion radiofrequency thermocoagulation in trigeminal neuralgia patients with multiple sclerosis

**DOI:** 10.55730/1300-0144.6139

**Published:** 2025-10-26

**Authors:** Emel BAŞAR, Nevcihan ŞAHUTOĞLU BAL, Erkan Yavuz AKÇABOY

**Affiliations:** Department of Pain Medicine, Ankara Bilkent City Hospital, Ankara, Turkiye

**Keywords:** Gasserian ganglion block, trigeminal neuralgia, multiple sclerosis

## Abstract

**Background/aim:**

Trigeminal neuralgia (TN) secondary to multiple sclerosis (MS) is a rare but debilitating condition. Aside from two case reports, there are limited data on the use of Gasserian ganglion radiofrequency interventions in this patient population. This study aims to evaluate the outcomes of combined radiofrequency thermocoagulation (RFT) and pulsed radiofrequency (PRF) treatment targeting the Gasserian ganglion in patients with MS-related TN.

**Materials and methods:**

We retrospectively analyzed 12 patients with MS-associated TN who underwent combined Gasserian ganglion RFT and PRF treatment between 1 January 2021 and 1 January 2025. Patients were followed for six months. Visual analog scale (VAS) scores, treatment efficacy, and adverse events were recorded.

**Results:**

The mean age was 55.2 ± 7.7 years, with a female predominance (58.3%). The average MS duration was 16.4 ± 4.6 years; one-third of patients had relapsing–remitting MS. TN was more commonly left-sided (58.3%). Interventions targeted V2 (16.7%), V3 (41.7%), or both branches (41.7%). At six months, all patients experienced >50% pain relief; sustained relief was observed in 75% of patients at 12 months.

**Conclusions:**

To our knowledge, this may represent one of the most extensive retrospective case series evaluating the combined use of RFT and PRF in patients with MS-related TN. The treatment was found to be effective and well-tolerated, with no significant procedure-related complications observed. While the results are encouraging, further prospective studies with larger sample sizes are warranted to validate these findings.

## 1. Introduction

Trigeminal neuralgia (TN) is a neuropathic pain condition characterized by sudden, sharp, electric shock-like facial pain episodes affecting one or more branches of the trigeminal nerve [1].

TN is typically categorized into three types based on etiology: classical, symptomatic, and idiopathic. Classical TN accounts for the majority of cases (approximately 85%) and is most often caused by vascular compression (commonly by the superior cerebellar artery) at the root entry zone of the trigeminal nerve [2]. In some cases, venous compression may also be implicated. Symptomatic TN, comprising 10–15% of cases, is associated with underlying neurological pathologies such as multiple sclerosis (MS), brainstem infarction, or space-occupying lesions such as cerebellopontine angle tumors and metastases.

Among these, MS is a well-recognized contributor to TN, increasing the risk up to 20-fold, with a reported prevalence of approximately 4% among MS patients [3].

TN associated with MS (MS–TN) tends to occur at a younger age, frequently exhibits bilateral involvement (in ~18% of cases), and is often accompanied by sensory abnormalities. MS itself is a chronic autoimmune disorder characterized by inflammatory demyelination and neurodegeneration affecting the central nervous system [4].

Increasing evidence suggests that focal demyelination at the trigeminal root entry zone, where central and peripheral myelin systems meet, may be a shared pathological mechanism underlying both classical and MS-related TN [5].

While carbamazepine remains a first-line treatment for TN, its efficacy is often reduced in MS-related cases. Moreover, MS patients may experience additional neurological symptoms such as gait disturbances, muscle weakness, and dizziness. For drug-resistant TN, a range of interventional options is available, including microvascular decompression, stereotactic radiosurgery, and percutaneous procedures targeting the Gasserian ganglion [4]. Despite advances, the optimal management of MS–TN remains challenging, warranting further investigation into effective and safe treatment modalities.

## 2. Materials and methods

We retrospectively reviewed the medical records of 12 patients diagnosed with TN secondary to MS, who underwent combined Gasserian ganglion radiofrequency thermocoagulation (RFT) and PRF therapy at our clinic between 1 January 2021 and 1 January 2025. The study was approved by the Ankara Bilkent City Hospital Ethics Committee (935-19.02.2025), and written informed consent was obtained from all participants. The study adhered to the principles of the Declaration of Helsinki.

Patients were followed for six months postprocedure, during which time visual analog scale (VAS) scores, treatment efficacy, and adverse effects were assessed. For optimal visualization during the procedure, patients were positioned supine with their heads secured with tape. A posteroanterior (PA) fluoroscopic view was used to orient the C-arm caudally, and the tilt was adjusted to achieve a submental view. The foramen ovale was identified using an anterior–posterior oblique view.

Under sterile conditions and after local infiltration with 2% prilocaine (Pricain 2%, 20 mL; Polifarma, Turkey), the trigeminal ganglion block was initiated by inserting a needle 2–3 cm lateral to the mouth corner. Sedation was administered with propofol and midazolam. A 22-gauge radiofrequency cannula (10 cm in length, with a 5-mm active tip) was advanced under fluoroscopy through the foramen ovale to target the mandibular branch (V3) of the trigeminal nerve. Masseter muscle twitches at 2 Hz stimulation confirmed ganglion engagement. The needle was further advanced to elicit paresthesia in the desired branch at 50 Hz stimulation, with voltage up to 1 V. Upon reaching the maxillary branch (V2), masseter twitches ceased.

The procedure began with PRF at 42 °C, 50 Hz, and 1 V for 120 s, followed by sequential RFT lesioning at 65 °C, 70 °C, and 75 °C, each for 60 seconds. PRF was reapplied at the end of the procedure. All patients tolerated the intervention well without significant discomfort. Treatments were performed using a NeuroTherm NT1100 radiofrequency generator (Abbott Medical, USA), which allows precise lesioning and effective pain control with minimal complications.

Data analysis was conducted using SPSS version 27.0 (IBM Corp., Armonk, NY). Normality of numerical variables was assessed using the Shapiro–Wilk test, histograms, and Q–Q plots. Normally distributed data are presented as mean ± standard deviation, while nonnormally distributed data are reported as median and interquartile range (IQR) or median (minimum–maximum). Categorical variables are expressed as frequencies and percentages.

## 3. Results

The demographic and clinical features of the patients with MS are given in Table 1. The mean age was 55.2 ± 7.7 years, with a female predominance of 58.3% (7/12). The average duration of MS was 16.4 ± 4.6 years. One-third (4/12) of the patients had the relapsing–remitting MS (RRMS) subtype. TN was more frequently left-sided (58.3%, 7/12) than right-sided (41.7%, 5/12).

**Figure 1:**
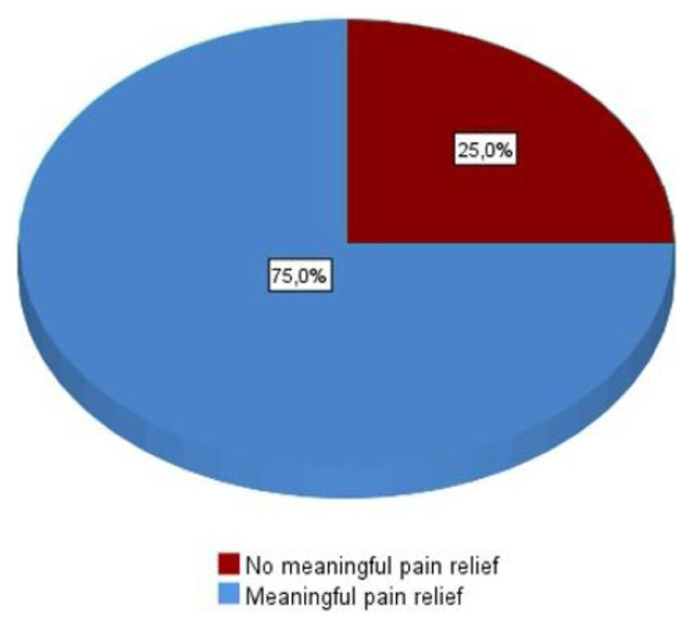
This figure shows meaningful pain relief at the twelfth month.

Regarding medication, 11 patients (91.7%) were on a combination of carbamazepine and gabapentinoids, while one patient (8.3%) was receiving carbamazepine alone. Interventions included V2 block in 2 patients (16.7%), V3 block in five patients (41.7%), and combined V2–V3 block in 5 patients (41.7%). At six months posttreatment, all patients (100%) reported meaningful pain relief, defined as greater than 50% reduction in pain scores, which decreased to 75% at 12 months (Figure 1). A second RFT session was performed in five patients (41.7%) at a mean interval of 17.7 ± 7.7 months following the initial procedure. Additionally, two patients (16.7%) underwent a third RFT session eight and nine months after the second treatment.

**Figure 2:**
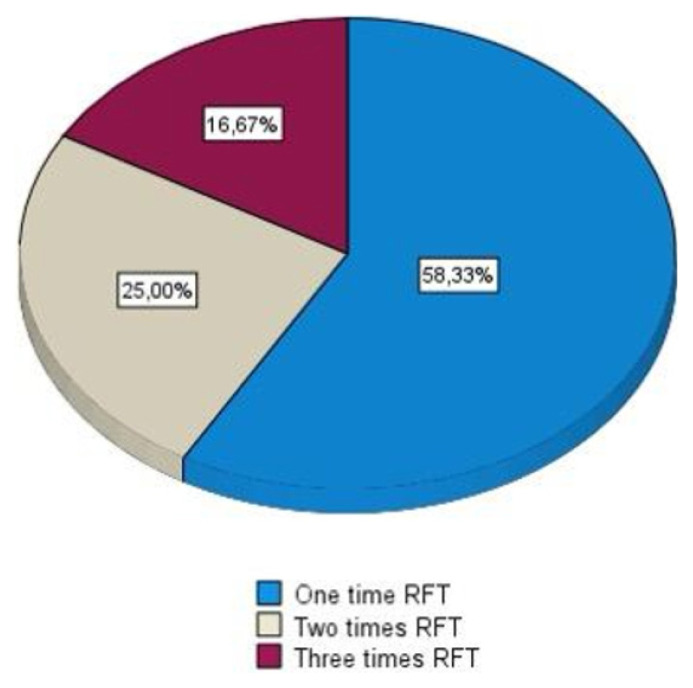
This figure shows the number of radiofrequency treatment protocols.

Detailed pain scores and follow-up data are presented in Table 2, while the distribution of RFT session frequencies is illustrated in Figure 2. Importantly, no complications or adverse events related to the procedure were observed in any patient.

## 4. Discussion

This retrospective study evaluated the combined use of fluoroscopy-guided RFT and PRF targeting the Gasserian ganglion in patients with TN secondary to MS. Our findings suggest that this combined approach offers effective and sustained pain relief, with 100% of patients achieving >50% pain reduction at 6 months and 75% maintaining this benefit at 12 months. Notably, no serious complications were observed.

In patients with refractory TN who cannot tolerate or fail to respond adequately to pharmacologic treatments such as carbamazepine or oxcarbazepine, interventional options are warranted. Established procedures include microvascular decompression (MVD), percutaneous balloon compression, gamma knife radiosurgery, and Gasserian ganglion interventions such as RFT and PRF [6–8].

RFT involves continuous, high-frequency current application, producing thermal lesions that disrupt nociceptive transmission by destroying nerve fibers [6]. In contrast, PRF delivers energy at subneuroablative temperatures (<42°C), preserving tissue integrity while modulating pain pathways [7,8]. Proposed mechanisms of PRF include suppression of C-fos expression [9,10], antiallodynic effects [11], attenuation of neuroinflammation, and support for neuronal recovery [12].

Previous studies have examined the combination of RFT and PRF to mitigate complications associated with high-temperature RFT. For example, Zhao et al. compared RFT at 70 °C and 75 °C, with or without adjunctive PRF, and found that combined therapy may reduce postoperative complications such as facial numbness and masticatory weakness. However, pain outcomes were similar across groups [13]. Our results are consistent with these findings, demonstrating that the combined technique is safe even at higher temperatures (up to 75 °C) without significant adverse effects. Similarly, Eissa et al. applied a dual PRF+RFT protocol at lower temperatures (60–65 °C) to reduce neurodestructive effects. While their approach minimized complications, they reported no cases of complete pain relief [14]. In contrast, our higher-temperature approach (75 °C) yielded complete pain resolution in 33.3% of patients at six months, suggesting a potential trade-off between lesion size and treatment efficacy. However, larger studies are needed to confirm this.

Supporting the hypothesis that demyelination plays a key role in MS-related TN, Plantone et al. reported pontine lesions and cerebrospinal fluid oligoclonal bands indicative of MS [15]. In our cohort, 50% of patients exhibited pontine demyelinating lesions on MRI, reinforcing the involvement of central demyelination in TN pathogenesis among MS patients.

Our findings are consistent with the recent study by Sözer et al., which retrospectively analyzed 42 patients with MS-related TN treated with radiofrequency ablation (RFT), gamma knife radiosurgery (GKRS), or microvascular decompression (MVD). Similar to their results, we observed that RF provides superior immediate pain relief and greater reduction in VAS scores than GKRS. Notably, Sözer et al. reported no significant differences in relapse-free survival or time to repeat procedures between RFT and GKRS modalities, paralleling our observation of sustained pain relief at 6 and 12 months following combined RFT and PRF treatment. Their data further indicated that the presence of demyelinating plaques at the brainstem or root entry zone did not significantly affect the total number of procedures required when RFT was the initial treatment, suggesting that RFT may offer a therapeutic advantage in managing more refractory cases of MSrTGN [16]. While our study focused on a combined RFT and PRF approach targeting the Gasserian ganglion, and theirs included additional modalities such as GKRS and MVD, both studies highlight the importance of individualized treatment selection tailored to patient-specific clinical profiles and disease characteristics. The congruence of these findings reinforces the validity of RF-based interventions as a valuable component in the multimodal management of MS-related TN.

The literature on the combined use of RFT and PRF in MS-related TN remains limited. Apart from two case reports by Chakole et al. and Tamura et al., no larger series have been reported. Chakole et al. described a single case with successful pain relief following sequential temperature RFT [17]. Tamura et al. treated a patient with bilateral TN using low-temperature PRF and RFT, achieving long-term bilateral pain relief with high patient satisfaction [18]. Compared with these isolated reports, our study provides the most extensive known case series to date on this topic and includes longer-term follow-up data (12 months).

While our findings are promising, this study’s limitations include its retrospective design and small sample size. Furthermore, the absence of a control group limits direct comparisons with alternative treatment strategies. Future prospective, randomized studies with larger populations are needed to validate our results and explore optimal RFT/PRF parameters in this unique patient population.

## 5. Conclusion

In conclusion, our treatment protocol, comprising PRF at 42 °C for 120 s, followed by sequential RFT at 65 °C, 70 °C, and 75 °C for 60 seconds each, with a final 120-s PRF application, proved to be an effective and safe option for managing TN associated with MS.

This combined approach offers a promising therapeutic alternative for MS patients suffering from TN, providing substantial and durable pain relief with minimal complications.

## Figures and Tables

**Table 1:** Baseline demographic and clinical features of the patients (n=12).

No	Age	Gender	Type of MS	MS duration (yr.)	MS medications	Demyelination in pons on MRI	Pain side	Pain location
1	64	F	RRMS	25	Ocrelizumab	yes	left	V2
2	60	M	RRMS	9	Dimetilfumarat	no	left	V3
3	55	F	PMS	20	Ocrelizumab	no	left	V2 and V3
4	63	F	RRMS	20	Fİngolimod	no	right	V3
5	47	F	RRMS	12	Dimetilfumarat	yes	left	V2 and V3
6	43	M	RRMS	15	Fingolimod	yes	right	V3
7	50	F	PMS	13	Fingolimod	yes	right	V2
8	46	M	RRMS	15	Kladribin	no	left	V2 and V3
9	58	M	SPMS	20	Ocrelizumab	yes	right	V2 and V3
10	57	M	RRMS	15	Teriflunomide	yes	left	V3
11	67	F	PMS	13	Fingolimod	no	left	V3
12	52	F	RRMS	20	Fingolimod	no	right	V2 and V3

MS: Multiple sclerosis; RRMS: Relapsing–remitting multiple sclerosis; PMS: Progressive multiple sclerosis; SPMS: Secondary progressive multiple sclerosis; VAS: Visual analog scale

**Table 2:** Pain scores at follow-up for all patients with and without repeated trigeminal nerve radiofrequency treatment protocol (RFTP) (n = 12).

No	VAS-baseline	VAS-6th mo	VAS-12th mo	Time to second TN RFTP (mo)	VAS-before second TN RFTP	VAS-6th mo after the second TN RFTP	VAS-12th mo after the second TN RFTP	Time to third TN RFTP (mo)	VAS-before third TN RFTP	VAS-6th mo after the third TN RFTP	VAS-12th mo after the third TN RFTP
1	9	1	1	–	–	–	–	–	–	–	–
2	8	0	2	–	–	–	–	–	–	–	–
3	9	1	2	7	8	2	1	8	8	1	2
4	7	2	2	–	–	–	–	–	–	–	–
5	9	3	3	24	9	0	2	–	–	–	–
6	9	0	7	12	7	0	2	–	–	–	–
7	8	1	1	–	–	–	–	–	–	–	–
8	9	3	5	–	–	–	–	–	–	–	–
9	8	0	2	24	8	0	2	–	–	–	–
10	9	2	5	–	–	–	–	–	–	–	–
11	9	1	2	–	–	–	–	–	–	–	–
12	10	0	3	21	8	0	0	9	7	0	1

VAS: Visual analog scale; TN: Trigeminal nerve; RFTP: Radiofrequency treatment protocol
